# Adenovirus-mediated interferon *α* gene transfer induces regional direct cytotoxicity and possible systemic immunity against pancreatic cancer

**DOI:** 10.1038/sj.bjc.6602713

**Published:** 2005-08-02

**Authors:** M Ohashi, K Yoshida, M Kushida, Y Miura, S Ohnami, Y Ikarashi, Y Kitade, T Yoshida, K Aoki

**Affiliations:** 1 Genetics Division, National Cancer Center Research Institute, 5-1-1 Tsukiji, Chuo-ku, Tokyo 104-0045, Japan; 2 Section for Studies on Host-immune Response, National Cancer Center Research Institute, 5-1-1 Tsukiji, Chuo-ku, Tokyo 104-0045, Japan; 3 Pharmacology Division, National Cancer Center Research Institute, 5-1-1 Tsukiji, Chuo-ku, Tokyo 104-0045, Japan; 4 Laboratory of Molecular Biochemistry, Department of Biomolecular Science, Faculty of Engineering, Gifu University, Yanagido 1-1, Gifu 501-1193, Japan

**Keywords:** gene transfer, pancreatic cancer, interferon alpha, natural killer cell

## Abstract

We previously demonstrated a characteristically high sensitivity of pancreatic cancer cells to interferon alpha (IFN-*α*) gene transfer, which induced a more prominent growth suppression and cell death in pancreatic cancer cells than in other types of cancers and normal cells. The IFN-*α* protein can exhibit both direct cytotoxicity and indirect immunological antitumour activity. Here, we dissected and examined the two mechanisms, taking advantage of the fact that IFN-*α* did not show any cross-species activity in its *in vivo* effect. When a human IFN-*α* adenovirus was injected into subcutaneous xenografts of human pancreatic cancer cells in nude mice, tumour growth was significantly suppressed due to cell death in an adenoviral dose-dependent manner. The IFN-*α* protein concentration was markedly increased in the injected subcutaneous tumour, but leakage of the potent cytokine into the systemic blood circulation was minimal. When a mouse IFN-*α* adenovirus was injected into the same subcutaneous tumour system, all mice showed significant tumour inhibition, an effect that was dependent on the indirect antitumour activities of IFN-*α*, notably a stimulation of natural killer cells. Moreover, in this case, tumour regression was observed not only for the injected subcutaneous tumours but also for the untreated tumours at distant sites. This study suggested that a local IFN-*α* gene therapy is a promising therapeutic strategy for pancreatic cancer, due to its dual mechanisms of antitumour activities and lack of significant toxicity.

Adenocarcinoma of the pancreas is, at present, one of the most intractable cancers, and despite recent advances of therapeutic and diagnostic modalities, the prognosis remains unimproved (Haller, 2003; Safioleas and Moulakakis, 2004). Patients with pancreatic cancer often succumb to local recurrence or metastatic spread (Akerele *et al*, 2003; Safioleas and Moulakakis, 2004). One major reason for the poor prognosis is that the cancer cells, even when they are small, have a high propensity to infiltrate to the surrounding tissues and metastasise to distant organs. Therefore, it is often necessary to consider pancreatic cancer as a systemic disease, and new therapeutic strategies that effectively target this cancer *in vivo*, are required (Okusaka and Kosuge, 2004; Yoshida *et al*, 2004).

The interferon alpha (IFN-*α*) protein is a cytokine with multiple biological activities that include antiviral activity, regulation of cell proliferation and differentiation and immunomodulation (Pfeffer *et al*, 1998). The cytokine has been used worldwide for the treatment of a variety of cancers including some haematological malignancies (hairy cell leukaemia, chronic myeloid leukaemia, some B- and T-cell lymphoma) and certain solid tumours such as melanoma, renal carcinoma and Kaposi's sarcoma (Gutterman, 1994; Pfeffer *et al*, 1998). However, clinical experiences with IFN protein therapy of other cancers, especially in the treatment of many solid tumours, have generally not been encouraging (Pfeffer *et al*, 1998). In the conventional regimen of IFN clinical trials, the recombinant IFN-*α* protein is systemically administrated through subcutaneous, intravenous or intramuscular routes. However, pharmacokinetic studies have indicated that the half-life of the IFN-*α* protein is 3 h in the blood circulation and only 0.01% of subcutaneously injected IFN-*α* can reach the target sites (Salmon *et al*, 1996; Suzuki *et al*, 2003), indicating that IFN-*α* protein delivery might be insufficient and/or at an unsustainable level in the tumour site. This may be the major reason why the previous trials based on the conventional IFN-*α* therapy failed to show an adequate antitumour effect. By contrast, improved therapeutic effect and safety are expected for IFN-*α* gene transfer, because it allows an increased and sustained local concentration of this cytokine in the target sites while minimising unnecessary systemic distribution (Suzuki *et al*, 2003; Hatanaka *et al*, 2004). In fact, it has been reported that direct IFN-*α* gene transfer into tumours suppressed growth of various cancers such as breast cancer, prostate cancer, renal cancer, hepatocellular carcinoma, basal cell carcinoma and leukaemia (Zhang *et al*, 1996; Coleman *et al*, 1998; Hottiger *et al*, 1999; Iqbal Ahmed *et al*, 2001). Although no study of IFN-*α* gene transfer against pancreatic cancer had been reported, we recently found that the expression of IFN-*α* effectively induced growth suppression and cell death in pancreatic cancer cells, an effect that appeared to be more prominent than in other types of cancers and normal cells (Hatanaka *et al*, 2004).

In addition to its direct cytotoxicity, IFNs can also exhibit an indirect antitumour effect through immune stimulations. They induce a major histocompatibility complex class I expression, cause an increase in cytotoxic T-lymphocyte activity, enhance the generation of T helper cells and activate macrophages and natural killer (NK) cells (Chawla-Sarkar *et al*, 2003). In this study, to explore the immunological antitumour activity of IFN-*α* gene therapy, we tested mouse IFN-*α* gene delivery into human pancreatic cancer xenograft tumours in nude mice. Although the mouse IFN-*α* did not show a direct antiproliferative effect against human pancreatic cancer cells *in vitro*, the mouse IFN-*α* gene transduction into pancreatic cancer xenografts showed significant inhibition of tumour growth, which was attributed to an IFN-*α*-induced stimulation of NK cells. This study together with our previous report showing a characteristically high sensitivity of pancreatic cancer cells to the direct cytotoxicity of IFN-*α* transgene expression (Hatanaka *et al*, 2004) suggests that IFN-*α* gene therapy is a promising therapeutic strategy for pancreatic cancer, due to its dual mechanisms of antitumour activities.

## MATERIALS AND METHODS

### Cell lines and recombinant adenovirus vectors

Human pancreatic cancer cell lines (Panc-1, AsPC-1, BxPC-3 and MIAPaCa-2) and murine renal cancer cell line (Renca) were used in this study. All cell lines were obtained from American Tissue Culture Collection (Rockville, MD, USA), and were maintained in an RPMI-1640 medium with 10% foetal bovine serum. The recombinant adenovirus vectors expressing human IFN-*α*2a (AxCA-IFN), mouse IFN-*α* (AdCA-mIFN), enhanced green fluorescein protein (AdCA-EGFP) and alkaline phosphatase cDNA (AdCA-AP) were prepared as described (Aoki *et al*, 1999; Nakano *et al*, 2001; Suzuki *et al*, 2003). A cesium chloride-purified virus was desalted using a sterile Bio-Gel P-6 DG chromatography column (Econopac DG 10, BioRad, Hercules, CA, USA) and diluted for storage in a 13% glycerol/PBS solution. All viral preparations were confirmed to be free of E1^+^ adenovirus by PCR assay (Zhang *et al*, 1995).

### *In vitro* growth analysis

The AsPC-1 and Renca cells were seeded at 2 × 10^3^ well^−1^ in 96-well plates and infected with AxCA-IFN, AdCA-mIFN or AdCA-EGFP at an MOI of 10, 30 and 100. The cell numbers were assessed by a colorimetric cell viability assay using a water-soluble tetrazolium salt (Tetracolor One; Seikagaku Corp., Tokyo, Japan) 5 days after the infection. The absorbance was determined by spectrophotometry using a wavelength of 450 nm with 595 nm as a reference. The assays (carried out in eight wells) were repeated a minimum of two times and the mean±standard deviation (s.d.) was plotted. The data were expressed as the relative cell number (OD_450_ of AxCA-IFN- or AdCA-mIFN-infected cells/OD_450_ of AdCA-EGFP-infected cells).

### Animal model

Female, 7- to 8-week-old BALB/c nude mice were obtained from Charles River Japan (Kanagawa, Japan), and kept in a specific pathogen-free environment. Among the cell lines used in this study, we chose AsPC-1 for an *in vivo* gene transfer model, because the cells rapidly form a tumour when inoculated in the subcutaneous or peritoneal space of nude mice. For the direct intratumoral treatment of subcutaneous tumours, AsPC-1 cell suspensions (50 *μ*l of 5 × 10^6^ cells) were injected subcutaneously into the left leg. When the tumour mass was established (∼0.5 cm in diameter), 50 *μ*l of a viral solution was injected into the tumour with a 29-G hypodermic needle, and the animals were observed for tumour growth. The short (*r*) and long (*l*) diameters of the tumours were measured and the tumour volume of each was calculated as *r*^2^*l*/2. Tumour sizes (mean±s.e.m. (standard error of mean)) were measured on the days indicated. For the distant metastasis treatment, 5 × 10^6^ of AsPC-1 cells were injected subcutaneously into the left leg and 1 × 10^6^ of AsPC-1 cells were simultaneously injected into the right leg. When the tumour mass on the left leg was established (∼0.5 cm in diameter), 50 *μ*l (1.0 × 10^8^ PFU) of the viral solution was injected once into the subcutaneous tumour on the left leg, and the animals were observed for tumour growth on both legs. For the peritoneal dissemination treatment, 5 × 10^6^ cells of AsPC-1 cell suspensions were injected subcutaneously into the left leg. When the tumour mass on the left leg was established, 1 × 10^6^ or 5 × 10^6^ cells of AsPC-1 cell suspensions were injected into the peritoneal cavity. After 1 day, 1.0 × 10^8^ PFU of the viral solution was injected once into the subcutaneous tumour, and the animals were observed for survival. For NK cell depletion experiments, we started the intraperitoneal injection (50 *μ*l) of anti-asialo GM1 antibody (30 mg ml^−1^: Wako Chemicals USA Inc., Richmond, VA, USA) 2 days before the vector administration and the treatment was repeated every 2–3 days for five times. Animal protocols were reviewed and approved by the Institutional Animal Core and Use Committee of the National Cancer Center Research Institute.

### RT–PCR and flow cytometric analyses of NKG2D ligands

PCR amplification of five known genes of NKG2D ligands (MICA, MICB, ULBP-1, ULBP-2 and ULBP-3) and glyceraldehyde-3-phosphate dehydrogenase (GAPDH) was carried out using total RNA from the cells in a 50 *μ*l of PCR mixture containing 1.5 mM MgCl_2_, 0.2 mM dNTPs, 1 U of recombinant *Taq* DNA polymerase and the following primer set: MICA upstream (5′-TTCCTGCTTCTGGCTGGCATC-3′) and downstream primers (5′-GCAGAAACATGGAATGTCTGCCAA-3′); MICB upstream (5′-CTGCTGTTTCTGGCCGTCGC-3′) and downstream primers (5′-GAAACATATGGAAAGTCTGTCCG-3′); ULBP-1 upstream (5′-CGCCTTCCTTCTGTGCCTCC-3′) and downstream primers (5′-AGATGATGAGAAGGCTCCAGGG-3′); ULBP-2 upstream (5′-AAGATCCTTCTGTGCCTCCCG-3′) and downstream primers (5′-GGATGATGAGGAGGCAGCAAAG-3′); ULBP-3 upstream (5′-GCGATCCTTCCGCGCCTC-3′) and downstream primers (5′-CAGGGTTTCTCGCTGAGGGAC-3′); and GAPDH upstream (5′-TGAAGGTCGGAGTCAACGGATTTGGT-3′) and downstream primers (5′-CATGTGGGCCATGAGGTCCACCAC-3′). In total, 35 cycles (GAPDH: 27 cycles) of the PCR were carried out at 94°C for 30 s, 68°C (MICB: 65°C, GAPDH: 60°C) for 1 min and 72°C for 1 min. The PCR products were electrophoresed on a 2% agarose gel. Daudi (human Burkitt's lymphoma) and K562 (human chronic myelogenous leukaemia) cells were used as negative and positive controls, respectively, for NKG2D ligands. The surface expression of MICA and MICB was then evaluated by flow cytometry. Cells (5 × 10^5^) were incubated at 4°C for 30 min with phycoerythrin-conjugated mouse anti-human MICA/B monoclonal antibody (6D4; IgG, BD Biosciences Pharmingen, San Jose, CA, USA) or isotype control antibody (IgG), and then washed twice with PBS containing 2% BSA and 0.05% NaN_3_ and fixed with 2% paraformaldehyde solution. Flow cytometry was carried out using a FACScan system (Becton Dickinson).

### NK cell assay

To determine NK cell activity, BALB/c nude mice were killed 14 days after the administration of the vector. The spleen from each animal was pressed through a nylon mesh and the cells were suspended in DMEM containing 2.5 mM EDTA and 3 U ml^−1^ of heparin. The suspension was then washed twice with PBS, and incubated with anti-NK cell antibody (DX5)-coated magnetic microbeads (Miltenyi Biotec, Auburn, CA, USA) and passed through a separation column to isolate the NK cells. NK cell activity was determined using a chromium release assay described previously (Tada *et al*, 2001). Briefly, 1 × 10^6^ YAC-1 mouse lymphoma cells (American Tissue Culture Collection) were radiolabelled with 3.7 Mbq of sodium chromate (^51^Cr) (Amersham Pharmacia Biotech, Piscataway, NJ, USA) in 500 *μ*l of RPMI-1640 at 37°C for 2 h. The cells were then washed twice with PBS and diluted to 1 × 10^4^ cells 100 *μ*l^−1^. Effector cells (NK cells) were added at various effector/target ratios, and a final volume of 100 *μ*l was added to the wells of a U-shaped 96-well plate. The reaction was incubated at 37°C for 4 h. The 96-well plate was then centrifuged, and the ^51^Cr release from 100 *μ*l of supernatant was counted in a liquid scintillation counter. The percentage of cytotoxicity was determined by the formula: (experimental release−spontaneous release/maximum release−spontaneous release) × 100.

### Histology

At 5 days after the injection of 1.0 × 10^8^ PFU of viral solutions (AxCA-IFN, AdCA-mIFN and AdCA-AP), the mice were killed and subcutaneous tumours were collected. The tumours were fixed with formalin, and sections were stained with haematoxylin–eosin or processed for immunohistochemistry with anti-asialo GM1 antibody (Wako Chemicals USA Inc.) and the terminal deoxy-nucleotidyltransferase-mediated dUTP-digoxigenin nick-end-labelling (TUNEL) assay (ApopTag *in situ* apoptosis detection kit; Intergen Company, Purchase, NY, USA). For microvessel detection, the fresh tumour samples were embedded in OCT compound (Sakura Finetechnical Co., Tokyo, Japan) and sections were fixed with 4% paraformaldehyde and then processed for immunohistochemistry with anti-mouse CD31 antibody (MEC13.3; BD Biosciences Pharmingen). Immunostaining was performed using the streptavidin–biotin–peroxidase complex techniques (Nichirei, Tokyo, Japan). Parallel negative controls without primary antibodies were run in all cases. The sections were counter-stained with methylgreen. Microvessel areas were quantified by counting of hotspots in sections, and 10 representative fields for each tumour were counted.

### Statistical analysis

Comparative analysis of *in vivo* tumour volume and *in vitro* cell proliferation was performed by the Student's *t*-test; mouse survival was analysed by Kaplan–Meier survival plot followed by a log-rank (Mantel–Cox) test. Differences were considered statistically significant when *P*-value was <0.05.

## RESULTS

### Direct antitumour effect of human IFN-*α* gene transduction into AsPC-1 tumours

**Figure 1:**
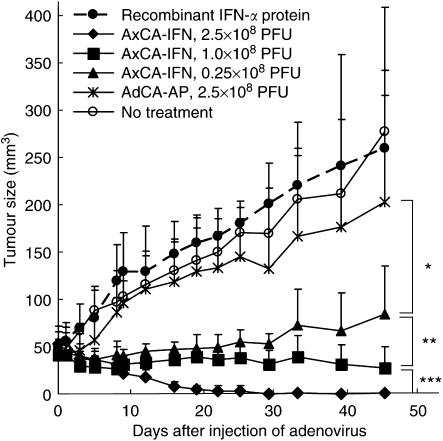
Direct antitumour effect of human IFN-*α* gene transduction into AsPC-1 subcutaneous tumours. When the AsPC-1 subcutaneous tumour was established on the left leg, the tumour was injected once with 2.5, 1.0 or 0.25 × 10^8^ PFU of AxCA-IFN, 2.5 × 10^8^ PFU of AdCA-AP or 3 × 10^5^ IU g^−1^ of recombinant IFN-*α* protein (*n*=6). Tumour size in each mouse was plotted on the days indicated. ^*^P<0.05 for tumours treated with AxCA-IFN at 0.25 × 10^8^ PFU *vs* treated with AdCA-AP at 2.5 × 10^8^ PFU, ^**^P<0.05 for tumours treated with AxCA-IFN at 0.25 × 10^8^ PFU *vs* treated at 1.0 × 10^8^ PFU, ^***^P<0.01 for tumours treated with AxCA-IFN at 1.0 × 10^8^ PFU *vs* treated at 2.5 × 10^8^ PFU.

**Figure 2:**
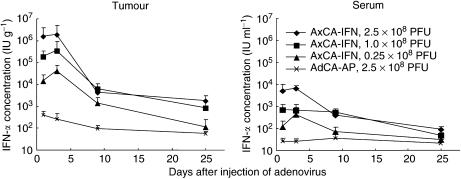
IFN-*α* concentration in the subcutaneous tumour and the serum after a single intratumoral injection of AxCA-IFN at various doses. IFN-*α* was measured by enzyme-linked immunosorbant assay (Immunotech, Marseille Cedex, France) (*n*=4).

We previously reported that an intratumoral injection of an adenoviral vector expressing the human IFN-*α* gene, AxCA-IFN (2.5 × 10^8^ PFU), effectively suppressed the growth of nude mouse subcutaneous tumours of the AsPC-1 human pancreatic cancer cells (Hatanaka *et al*, 2004). In this study, the superior antitumour effect of the IFN-*α* gene therapy over the recombinant IFN-*α* protein therapy was evaluated between the single injection of 1.0 × 10^8^ PFU of AxCA-IFN and 3 × 10^5^ IU g^−1^ of recombinant IFN-*α* protein, which corresponds to the top level achieved by the 1.0 × 10^8^ PFU of AxCA-IFN infection as shown in Figure 2. The injection of AxCA-IFN showed remarkable tumour suppressive effects in a dose-dependent manner, whereas recombinant IFN-*α* protein therapy failed to produce a significant therapeutic response (Figure 1), suggesting that an antitumour effect needs a sustained and efficient local expression of IFN-*α*.

### High IFN-*α* expression in subcutaneous tumour tissues following intratumoral injection of AxCA-IFN with little leakage into serum

To determine the kinetics of IFN-*α* expression after intratumoral injection of AxCA-IFN, the IFN-*α* levels were measured in the subcutaneous tumour and serum. As shown in Figure 2, the peak levels in the tumour tissue were observed 3 days after the injection and the expression continued for more than 10 days. Although the injection of 2.5 × 10^8^ PFU of AdCA-AP showed a slight expression of IFN-*α* at day 1, which may be a naïve reaction in response to viral infection, the IFN-*α* expression returned to the base line (<300 IU g^−1^) by day 9. The subcutaneous tumour showed approximately 100–1000-fold higher IFN-*α* levels than the serum did (Figure 2), confirming a markedly increased IFN-*α* concentration in the subcutaneous tumour with little leakage into the serum (Hatanaka *et al*, 2004). After the intratumoral injection of recombinant IFN-*α* protein (3 × 10^5^ IU g^−1^), the concentration of IFN-*α* was 609±130 IU g^−1^ at day 1 and returned to the base line by day 3, which indicated the injected recombinant IFN-*α* protein was rapidly degraded in the tumour.

### Inhibition of peritoneal dissemination by AxCA-IFN

**Figure 3:**
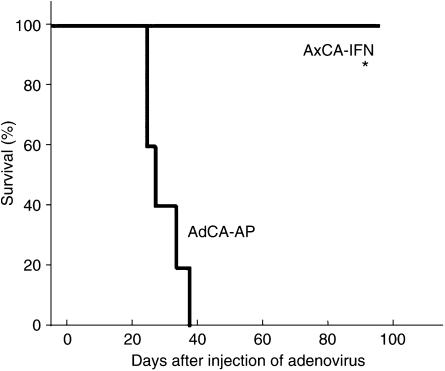
Treatment of peritoneal dissemination by the intraperitoneal injection of AxCA-IFN. At 3 days after the intraperitoneal injection of AsPC-1 cells (2 × 10^6^ cells), 2.5 × 10^8^ PFU of AxCA-IFN (*n*=5) or AdCA-AP (*n*=5) was intraperitoneally injected, and animals were observed for survival. ^*^P<0.01 for survival rates of mice treated with AxCA-IFN *vs* treated with AdCA-AP.

Peritoneal dissemination is an often fatal end-stage manifestation of pancreatic cancer in the clinical setting. To test the *in vivo* antitumour effect against peritoneal dissemination, 5 × 10^6^ of AsPC-1 cells were inoculated into the peritoneal cavity of nude mice, and 3 days later, the AxCA-IFN was injected intraperitoneally. Within 42 days after the adenovirus injection, all the control AdCA-AP-injected mice died of peritoneal dissemination that includes tumour formations on the mesentery, pancreas and hepatic hilus. By contrast, the AxCA-IFN-treated group showed a significant suppression of AsPC-1 cell growth in the peritoneal cavity, allowing all the mice to survive more than 100 days (Figure 3).

### No cross-species activity in the antiproliferative effect of IFN-*α*

**Figure 4:**
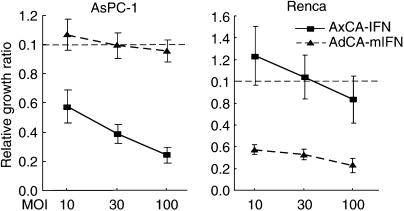
Lack of cross-species activity of human and mouse IFN-*α* gene transfer. AsPC-1 and Renca cells were infected with AxCA-IFN, AdCA-mIFN or AdCA-EGFP at an MOI of 10, 30 and 100. Cell growth was determined by cell proliferation assay 5 days after the infection.

To explore the immunological antitumour effect of IFN-*α* gene therapy, we transduced the mouse IFN-*α* gene into human pancreatic cancer xenografts in nude mice. First, the lack of cross-species activity of IFN-*α* was confirmed by infecting human pancreatic cancer (AsPC-1) and mouse renal cancer (Renca) cells *in vitro* with a human or mouse IFN-*α* adenovirus. The infection with AxCA-IFN showed significant growth suppression of AsPC-1 cells but not Renca cells. Conversely, the infection with AdCA-mIFN resulted in marked growth retardation in Renca cells, whereas its growth suppressive effect was not significant in AsPC-1 cells (Figure 4). In our previous report, IFN-*α* secreted into the medium appeared to account for most, if not all, of the growth suppression (Hatanaka *et al*, 2004).

### Expression of NKG2D ligands in human pancreatic cancer cells

**Figure 5:**
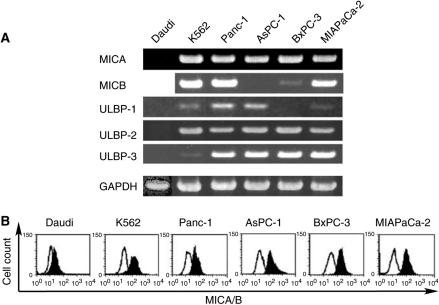
Expression of NKG2D ligands on pancreatic cancer cell lines. (**A**) RT–PCR analyses of NKG2D ligands. Total RNA was extracted from cell lines and after reverse transcription the mRNA of MICA, MICB, ULBP-1, ULBP-2 and ULBP-3 was detected by PCR. (**B**) Flow cytometric analysis of MICA and MICB. Cells were incubated with anti-human MICA/B monoclonal antibody (filled curve) or isotype control antibody (open curve), and flow cytometry was carried out using a FACScan system.

NK cells are an important element of the innate immune system as they are capable of killing tumour cells. The cytotoxic activity of NK cells is enhanced by IFN-*α*/*β* through TRAIL (tumour necrosis factor-related apoptosis-inducing ligand) gene induction (Sato *et al*, 2001). NKG2D is one of the activating receptors on NK cells, and a number of NKG2D ligands, such as MICA and MICB (major histocompatibility complex class I chain-related) and ULBP (UL16-binding proteins), have been identified (Groh *et al*, 1999; Bahram, 2000; Kubin *et al*, 2001; Stephens, 2001; Raulet, 2003; Yokoyama and Plougastel, 2003). It is known that the expression of NKG2D ligands is upregulated in transformed cells, and that the human NKG2D ligands can bind to mouse NKG2D, leading to the activation of mouse NK cells (Kubin *et al*, 2001). To examine whether the pancreatic cancer cells express the ligands, RT–PCR and flow cytometric analyses were performed in four cell lines (Panc-1, AsPC-1, BxPC-3 and MIAPaCa-2). As shown in Figure 5, all the pancreatic cancer cells examined expressed several kinds of NKG2D ligands, suggesting that the cancer cells are effective targets of NK cells.

### Immunological antitumour effect of mouse IFN-*α* gene transduction into AsPC-1 tumours

**Figure 6:**
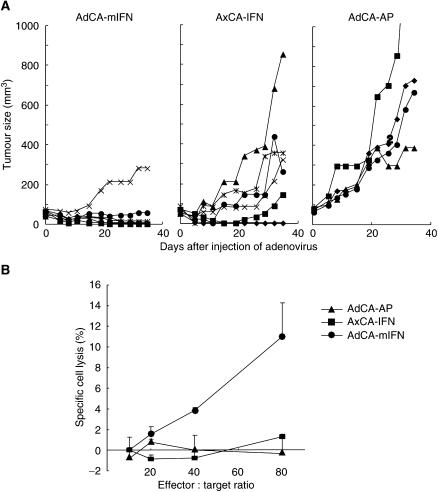
Immunological antitumour effect of mouse IFN-*α* gene transduction into AsPC-1 subcutaneous tumours in nude mice. (**A**) Growth inhibition of subcutaneous tumour injected with AxCA-IFN and AdCA-mIFN. When the AsPC-1 subcutaneous tumour was established on the left leg, 1.0 × 10^8^ PFU of viral solution was injected once into the tumour. Tumour size in each mouse (AdCA-mIFN: *n*=6; AxCA-IFN: *n*=6; AdCA-AP: *n*=4) was plotted on the days indicated. (**B**) NK cell activity of AdCA-mIFN-injected mice. NK cells were harvested from the spleens of treated animals 14 days after the intratumoral injection of adenoviruses.

We next examined whether mouse IFN-*α* gene therapy leads to regression of the human AsPC-1 xenograft tumours in nude mice. We injected subcutaneous AsPC-1 tumours with AdCA-mIFN, AxCA-IFN or AdCA-AP at a dose of 1.0 × 10^8^ PFU. Although AxCA-IFN effectively suppressed tumour growth as shown in our previous study (Hatanaka *et al*, 2004) and Figure 1, complete tumour regression was observed in only one of six mice, and in the particular experiment described here, one of the six mice failed to respond to AxCA-IFN (Figure 6A, middle panel). AdCA-mIFN caused complete regression in four of the six mice, and the remaining two showed a significant growth retardation (Figure 6A, left panel). There was no pathological change in major organs such as the brain, lung, heart, liver, spleen, pancreas, kidney, stomach, intestine, ovary and skeletal muscle of the vector-infected mice (data not shown).

**Figure 7:**
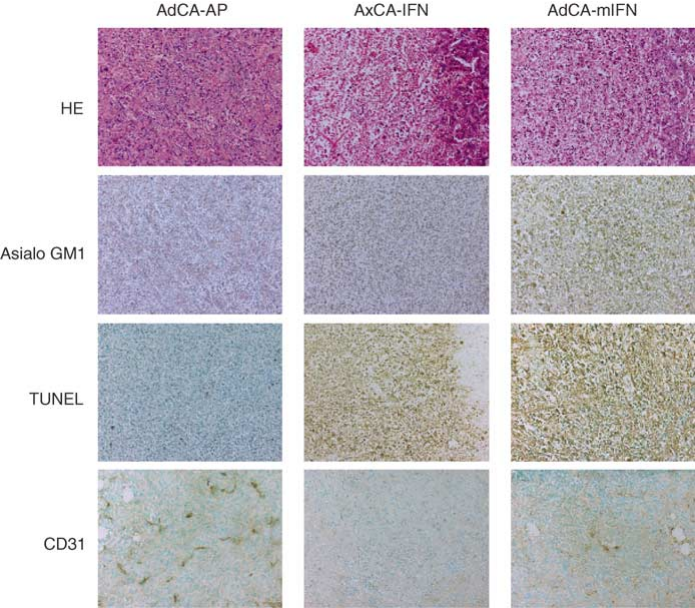
Histological and immunohistochemical analyses of AsPC-1 tumours treated with AxCA-IFN and AdCA-mIFN. At 5 days after the injection of 1.0 × 10^8^ PFU of viral solutions, the subcutaneous tumours were fixed with formalin for haematoxylin-eosin (HE) staining, immunohistochemistry with anti-asialo GM1 antibody or TUNEL staining (× 200). Fresh tumour samples were embedded in OCT compound and sections were fixed with 4% paraformaldehyde and then processed for immunohistochemistry with anti-mouse CD31 antibody (× 200).

To confirm that mouse IFN-*α* gene delivery enhanced NK cell activity, we collected splenocytes from the mice that received intratumoral AdCA-mIFN injection and examined the NK cell function. Increased NK cytolysis was observed in these mice compared with the AxCA-IFN- or AxCA-AP-injected mice (Figure 6B). Histological analysis of AdCA-mIFN-injected tumours revealed infiltration of many monocytic cells, which were stained with anti-asialo GM1 antibody, whereas AxCA-IFN-injected mice also showed massive cell death but rare infiltration of monocytic cells (Figure 7). TUNEL assay of AxCA-IFN- or AdCA-mIFN-injected tumours revealed marked apoptosis induction (Figure 7). An immunohistochemical staining with anti-murine CD31 antibody revealed a significantly decreased microvessel density in the IFN-*α* gene-transduced AsPC-1 subcutaneous tumour models (Figure 7); 10 representative fields under a microscope showed 121, 13 and 44 microvessel counts for the tumours injected with AdCA-AP, AxCA-IFN and AdCA-mIFN, respectively. However, it was not determined whether the loss of vessels was indirectly caused by the marked cell death or directly caused by the antiangiogenetic activity of IFN-*α*.

**Figure 8:**
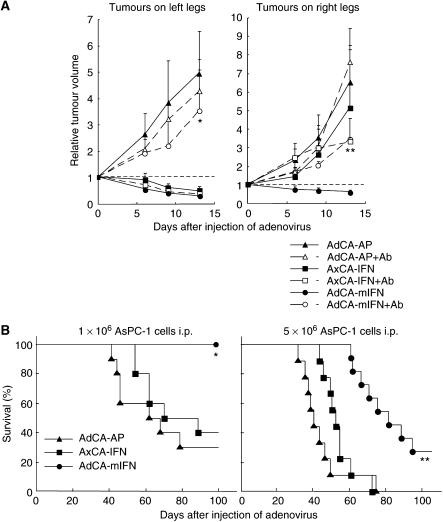
Suppression of tumours at distant sites by the intratumoral injection of AdCA-mIFN into subcutaneous xenografts. (**A**) Suppression of contralateral subcutaneous tumours. The AsPC-1 cells were injected subcutaneously into both legs, and then 1.0 × 10^8^ PFU of viral solution was injected once into the left tumour (*n*=6). The data (mean±s.d.) were expressed as the relative tumour volume, which is the tumour volume on the days indicated, divided by that on day 0. For NK cell depletion experiments, mice were treated with intraperitoneal (i.p.) injection of anti-asialo GM1 antibody for four times. ^*^P<0.05 for tumours on the left legs injected with AdCA-mIFN in the presence or absence of anti-asialo GM1 antibody, ^**^P<0.05 for tumours on the right legs untreated with AdCA-mIFN in the presence or absence of anti-asialo GM1antibody. (**B**) Suppression of peritoneal dissemination. When the AsPC-1 subcutaneous tumour was established on the left leg, 1 × 10^6^ (left panel) or 5 × 10^6^ cells (right panel) of AsPC-1 cell suspensions were injected into the peritoneal cavity, and 1 day later, 1.0 × 10^8^ PFU of viral solution was injected once into the subcutaneous tumour. (Left panel) AdCA-mIFN: *n*=10; AxCA-IFN: *n*=10; AdCA-AP: *n*=10. (Right panel) AdCA-mIFN: *n*=11; AxCA-IFN: *n*=9; AdCA-AP: *n*=9. ^***^P<0.01 for survival rate of AdCA-mIFN-treated mice *vs* AxCA-IFN- and AdCA-AP-treated mice.

Pretreatment with an anti-asialo GM1 antibody to purge NK cells reduced the antitumour effect of AdCA-mIFN (Figure 8A, left panel) significantly, suggesting that mouse IFN-*α* expression induced the activation of NK cells, which play a major role in the antitumour effect of AdCA-mIFN.

### No significant systemic toxicity after intratumoral injection of AdCA-mIFN

To characterise the potential toxicity of local IFN-*α* gene therapy, different doses of AdCA-mIFN were injected into the AsPC-1 subcutaneous tumours, and the animals were killed 5 or 11 days later to evaluate serum enzyme values and histology of major organs. All treated mice looked healthy during the course of the experiment. Although a mild elevation of the hepatic enzymes (AST and ALT) was observed at day 5 only when a relatively high dose (2.5 × 10^8^ PFU) of IFN-*α* adenovirus was injected into the tumours (Table 1), their values returned to a normal range at day 11. Other biochemical markers (albumin, alkaline phosphatase, BUN, creatinin) were within normal limits, and there were no pathological changes in major organs in all of the treated animals, suggesting that systemic toxicity of local IFN-*α* gene therapy is minimal.

### Suppression of tumours at distant sites by intratumoral injection of AdCA-mIFN

We then tested whether mouse IFN-*α* gene transduction has an effect on untreated tumours at distant sites. We injected AsPC-1 cells into both legs, and when the tumour diameter reached about 5 mm, 1 × 10^8^ PFU of AdCA-mIFN was injected once into the tumour on the left leg. A regression of both the treated tumour on the left leg and the untreated tumour on the opposite leg was observed, whereas the injection of the control AdCA-AP or AxCA-IFN did not affect the growth of tumours on the right leg. When we pretreated the mice with an anti-asialo GM1 antibody to purge NK cells, the mouse IFN-*α*-induced antitumour effect was cancelled (Figure 8A, right panel).

As another model of distant metastasis, we injected AsPC-1 cells into the left leg and the peritoneal cavity, and again, when tumour mass was established on the left leg, 1 × 10^8^ PFU of adenovirus was injected once into the tumour. The treatment with AdCA-mIFN resulted in a profound and statistically significant improvement (*P*<0.01) in the survival of the treated mice as compared with AxCA-IFN and AdCA-AP (Figure 8B). All of the dead animals were confirmed to have disseminated tumours in the peritoneal cavity. These data demonstrated that the antitumour effect of a local IFN-*α* gene therapy is not limited to a locally injected tumour site, but that it can induce a systemic immunity against pancreatic cancer cells.

## DISCUSSION

It has been reported that the modes of antitumour responses from IFN-*α* gene therapy manifested three aspects: direct antiproliferative effect (Zhang *et al*, 1996; Ahmed *et al*, 1999, 2001; Iqbal Ahmed *et al*, 2001; Demers *et al*, 2002), stimulation of cytotoxic T cells (Coleman *et al*, 1998; Horton *et al*, 1999; Li *et al*, 2001) and antiangiogenesis activity (Indraccolo *et al*, 2002a, 2002b). Although others highlighted each antitumour mechanism in the basic studies of IFN-*α* gene therapy, in this study we demonstrated that intratumoral injection of the IFN-*α* adenovirus could effectively suppress xenograft tumours of pancreatic cancer due to the dual mechanisms of antitumour activities: the direct regional apoptosis induction and the systemic immunological effect at least through NK cell activation. Furthermore, the IFN-*α* protein concentration was markedly increased in the injected subcutaneous tumour, but leakage into the serum was minimal, suggesting the safety of intratumoral injection of an IFN-*α* adenovirus. Qin *et al* (2001) also reported that IFN-*β* gene therapy induced direct antitumour activity and an immunological effect of NK cells against human glioblastoma. However, IFN-*β* protein showed less of an antiproliferative effect against pancreatic cancer cells than did IFN-*α* (unpublished data). Although the type 1 IFNs, the *α*-family and *β*, share receptor components, they were observed to have differences in their receptor binding and signalling (Abramovich *et al*, 1994a, 1994b), which may explain their different antitumour activities.

In this study, we injected the IFN-*α*-expressing vector locally to the tumour mass. In pancreatic cancer, such a regional therapy is particularly relevant, because locally advanced cases are surgically unresectable but can be accessible by laparoscopy-mediated injection or ultrasound- or CT-guided percutaneous injection. In a clinical setting, IFN-*α* gene therapy could exert tumour suppressive effects based both on direct cytotoxicity and indirect immunological antitumour activities, the combination of which is expected to be highly efficacious against locally advanced pancreatic cancer. Even though some pancreatic cancer cells are resistant to the direct antiproliferation and cell death-inducing activities of IFN-*α*, such resistant cancer cells could be suppressed by the activation of the innate immune system, as shown in this study. The NK cells can be activated locally in the tumour and/or activated systemically through immune organs such as the spleen (Qin *et al*, 2001). The latter can result from vector dissemination into the spleen or the circulation of a low level of IFN-*α* that is released by the vector-transduced tumour cells. Pancreatic cancer cells might be an effective target of activated NK cells, because the cancer cell lines express a variety of NKG2D ligands as shown in Figure 5. Moreover, cytokine-mediated activation of a host adaptive immune system may also contribute to the mounting of a systemic immunity against pancreatic cancer in this direct local IFN-*α* gene therapy, although this effect could not be evaluated in our nude mice study. In addition to locally advanced cases, distant metastasis at the liver and in the peritoneal cavity may be a clinical target of local IFN-*α* gene therapy.

With respect to the safety of the therapy, it is noteworthy that the IFN-*α* gene transfer showed very limited antiproliferative effects on normal cells such as hepatocytes and vascular endothelial cells (Hatanaka *et al*, 2004), although their mechanisms are still not fully understood. In addition, there was much difference in the IFN-*α* protein concentration between a vector-injected tumour tissue and the serum. In fact, although a mild elevation of hepatic enzymes was observed at day 5 only when a relatively high dose (2.5 × 10^8^ PFU) of IFN-*α* adenovirus was injected into the tumours (Table 1), the elevation returned to a normal range at day 11. Therefore, we expect that the toxicity of local IFN-*α* gene therapy is tolerable in human clinical trials, although more preclinical toxicity study using larger animals such as non-human primates will be required. One additional potential advantage of gene therapy technology is that we can design and preinstall safety devices to control transgene effects, such as suicidal gene, regulatable promoter or a Cre/loxP-mediated shut-off system of IFN-*α* expression *in vivo* (Suzuki *et al*, 2003). In sum, our preclinical study suggests that the regional adenovirus-mediated gene transfer of IFN-*α* is one of the promising new approaches to the pancreatic cancer. The strategy may deserve an evaluation in a future clinical trial for this highly intractable cancer.

## Figures and Tables

**Table 1:** Evaluation of selected serum chemistries 5 days after intratumoral injection of AdCA-mIFN

	**AdCA-mIFN**		**AdCA-AP**		**No treatment**
**Dose (PFU): × 10^8^**	**2.5**	**1.0**	**0.25**	**2.5**	**—**
*Mouse IFN concentration*					
Tumour (pg g^−1^): × 10^3^	252.3±292.2	53.0±30.0	14.3±13.4	1.4±0.6	0.8±0.2
Serum (pg ml^−1^): × 10^3^	1.7±0.9	0.3±0.1	0.3±0.2	0.2±0.1	0.2±0.1
					
Albumin (g dl^−1^)	2.8±0.3	2.9±0.4	2.8±0.4	2.7±0.1	2.9±0.3
Alk. phos. (IU l^−1^)	231±71	186±29	199±44	186±21	218±81
Amylase (U l^−1^)	13 910±3932	16 595±6937	17 790±453	14 850±353	10 269±3922
BUN (mg dl^−1^)	19.1±1.6	23.6±6.4	21.2±2.4	22.7±1.3	23.9±4.0
Creatinine (mg dl^−1^)	0.08± 0.02	0.09± 0.02	0.1±0.01	0.09±0.02	0.11±0.03
LDH (IU l^−1^)	379± 69	316±68	560±244	486±252	368±216
AST (IU l^−1^)	126±4	101±42	93±34	68±17	70±23
ALT (IU l^−1^)	54±32	28±4	25±7	18±10	19±5
T. bil. (mg dl^−1^)	0.05±0.01	0.04±0.01	0.05±0.01	0.06±0.01	0.04±0.01
